# Advanced Glycation End Products Induce Vascular Smooth Muscle Cell-Derived Foam Cell Formation and Transdifferentiate to a Macrophage-Like State

**DOI:** 10.1155/2020/6850187

**Published:** 2020-08-07

**Authors:** Zhengyang Bao, Lihua Li, Yue Geng, Jinchuan Yan, Zhiyin Dai, Chen Shao, Zhen Sun, Lele Jing, Qiwen Pang, Lili Zhang, Xiaodong Wang, Zhongqun Wang

**Affiliations:** ^1^Department of Cardiology, Affiliated Hospital of Jiangsu University, Zhenjiang 212001, China; ^2^Department of Pathology, Affiliated Hospital of Jiangsu University, Zhenjiang 212001, China

## Abstract

**Background:**

Advanced glycation end products play an important role in diabetic atherosclerosis. The effects of advanced glycation end products (AGEs) on vascular smooth muscle cell- (VSMC-) derived foam cell formation and phenotypic transformation are unknown.

**Methods:**

Serological and histological samples were obtained from diabetic amputation patients and accident amputation patients from the Affiliated Hospital of Jiangsu University. CD68/Actin Alpha 2 (ACTA2) coimmunofluorescence sections were used to quantify the number of VSMCs with macrophage-like phenotypes. Western blotting was used to detect the expression of the receptor of advanced glycation end products in vascular samples. Enzyme-linked immunosorbent assay (ELISA) was used to evaluate the level of serum N*ε*-carboxymethyl-lysine (CML). In vitro oil red O staining was used to examine lipid accumulation in VSMCs stimulated by CML. The expression of VSMCs and macrophage markers was measured by western blotting and quantitative real-time PCR. Furthermore, changes in VSMC migration and secretion were detected by the Transwell assay and ELISA.

**Results:**

In the arterial plaque sections of diabetic patients, VSMCs transformed to a macrophage-like phenotype. The serum CML and RAGE levels in the plaques were significantly higher in the diabetes group than those in the healthy control group and were significantly related to the number of macrophage-like VSMCs. CML stimulation promoted intracellular lipid accumulation. However, CML stimulation decreased the expression of VSMC markers and increased the expression of macrophage phenotype markers. Finally, CML promoted smooth muscle cell migration and the secretion of proinflammatory-related factors.

**Conclusions:**

CML induces VSMC-derived foam cell formation, and VSMCs transdifferentiate to a macrophage-like state, which may be mediated by the activation of RAGE.

## 1. Background

Atherosclerosis is characterized by a low degree of sterile inflammation of the arterial wall. The main pathological manifestations include endothelial cell injury and dysfunction [1], foam cell formation [2], and phenotypic transformation and calcification of VSMCs [3, 4]. During the progression of the arteriosclerotic process, most of the foam cells in the plaque are derived from circulating monocytes, which are recruited from the intima and stimulated by proinflammatory cytokines secreted by damaged endothelial cells [5]. However, in mouse models, macrophages are reduced by targeting or natural interference (possibly due to migration or apoptosis) as atherosclerosis progresses [6]. Although the proportion of macrophages in the plaques is gradually reduced, macrophage markers are detected. Because this phenomenon may be due to the recruitment of mononuclear macrophages and redundancy of retention factors, researchers have turned their attention to other cells capable of transforming into macrophages, such as endothelial cells, smooth muscle cells, and progenitor cells, the most important of which are VSMCs [7, 8].

VSMCs are not terminally differentiated and exhibit phenotypic plasticity. Under steady-state conditions, intravascular smooth muscle cells rarely proliferate and exhibit low levels of synthetic activity [3]. Under in vitro culture conditions or during atherosclerosis, smooth muscle cells undergo a phenotypic transformation: in vitro, the ability to proliferate, migrate, and secrete various extracellular matrices and cytokines is increased [9]. Recent studies have found that VSMCs are another important source of foam cells, accounting for 40%-50% of the total foam cell population, and gradually increase in proportion to disease progression [7, 8].

Cholesterol-loaded smooth muscle cells exhibit decreased expression of contractile phenotype-specific genes and significantly increased macrophage marker expression but do not exhibit intact macrophage function, such as innate immune signalling, phagocytosis, and efferocytosis [10].

Diabetic patients have an earlier onset of cardiovascular disease (approximately 14.6 years) than nondiabetic patients [11, 12], and the incidence is more diffuse. By accelerating the formation and progression of atherosclerotic lesions, the risk of cardiovascular disease is increased 2- to 4-fold [13, 14]. AGEs are modifications of proteins, lipids, and nucleic acids via a nonenzymatic reaction called glycation. Early glycation and oxidation processes result in the formation of Schiff bases and Amadori products. Further glycation of proteins and lipids causes molecular rearrangements that lead to the generation of AGEs. Previous studies have reported that, in hyperglycaemic environments and ageing environments formed in vivo, the advanced glycation end products bind to various receptors on blood vessel walls and affect extracellular and intracellular functions, promoting macrovascular and microvascular complications in diabetes [15].

The key active component of advanced glycation end products, N*ε*-carboxymethyllysine, promotes macrophage expression of CD36 and scavenger receptor class B type 1 (SR-B1) and induces lipid accumulation and foam cell formation, accelerating the progression of atherosclerosis [16]. Recent reports have indicated that receptor of advanced glycation end products (RAGE) modifies the cellular responses to enzyme-modified nonoxidative LDL (ELDL) by the upregulation of LOX-1 and uptake of oxLDL primed by ELDL in a RAGE-dependent manner [17], and advanced glycation end products and SMCs are the sources of foam cell formation. The relationship between inflammation and foam cell formation has not been clarified. In this study, our present data suggest that advanced glycation end products induce lipid accumulation in VSMCs and transdifferentiate VSMC to a macrophage-like state.

## 2. Methods

### 2.1. Reagents

CML was purchased from PolyPeptide Laboratories (San Diego, USA). Oxidized low-density lipoprotein (oxLDL) was acquired from Yiyuan Biotechnology (Guangzhou, China). The CML ELISA kit was provided by Nanjing Jiancheng Bioengineering Institute (Nanjing, China). RAGE and galectin-3 siRNA were purchased from RiboBio Biotechnology (Guangzhou, China), smooth muscle (SM) antiactin was purchased from Sigma-Aldrich (St. Louis, MO, USA), and antibodies targeting RAGE, galectin-3, *β*-actin, and CD68 were obtained from Abcam (USA).

### 2.2. Patients

Anterior tibial arteries from human diabetic amputees (*n* = 16) and accident amputees (*n* = 20) were obtained from the Department of Orthopedics, the Affiliated Hospital of Jiangsu University (Zhenjiang, China), and were recruited from October 2019 to February 2020. All patients were treated with standard insulin therapy to control blood glucose before the operation. Data regarding age, sex, duration of diabetes mellitus, hypertension status, fasting plasma glucose (FPG), and lipid profile (total cholesterol, fasting triglycerides, low-density lipoprotein (LDL) cholesterol) were collected. Written informed consent was obtained from all the patients, the study was approved by the Ethics Committee of the Affiliated Hospital of Jiangsu University (SWYXLL20191119-6) and the Chinese Clinical Trial Registry (ChiECRCT20190206), and the study was conducted in agreement with the institutional guidelines.

### 2.3. Lipid Content and Immunostaining

Vascular samples from patients were embedded in an optimum cutting temperature (OCT) compound, frozen at -80°C, and cut into 5 *μ*m thick sections. Serial sections were stained with SM *α*-actin for 12 h and CD68 to determine the cell type of origin of the foam cells. Briefly, the slides were blocked with 10% normal donkey serum for one hour and then were incubated with monoclonal SM *α*-actin-FITC and CD 68 overnight. AlexaFluor® 555-conjugated donkey anti-rabbit IgG was used to detect CD68 by dual immunofluorescence staining. Nuclei were stained using DAPI.

### 2.4. Cell Culture

VSMCs were isolated from the aorta of male, 8-week-old C57BL/6J mice as reported previously. The mice were euthanised, and the chest was removed. The thoracic aorta was dissected under a surgical microscope. The aorta was removed and washed with PBS several times before treatment with type II collagenase. The vessels were then cut into pieces and cultured in cell culture flasks to obtain VSMCs, and passages 5–8 were used in the experiments.

### 2.5. RNA Interference

VSMCs were plated in 500 *μ*l of growth medium without antibiotics. The cells were transfected after reaching 30%~50% confluence. The procedures used for this experiment were similar to those previously described. The RAGE siRNA, LOX-1, and galectin-3 siRNA were synthesised by OriGene Technologies, Inc. (Rockville, MD, USA), and a nontargeting siRNA duplex sequence (universally scrambled) served as a negative control. Transfection was performed according to the manufacturer's instructions. Serum-starved quiescent VSMCs were subjected to cyclic stress in the presence and absence of AGEs. After transfection, the samples were collected for western blotting and immunofluorescence staining.

### 2.6. Oil Red O Staining of Foam Cells

Cultured VSMCs were plated on six-well plates and treated with targeted reagents for 72 h in serum-free DMED. Afterwards, the cells were washed twice with PBS, fixed for 20 min in 4% paraformaldehyde and stained for 30 min in oil red O working solution (3 parts oil red O stock solution: 2 parts distilled water), and washed 3 times in isopropanol. The stained samples were evaluated under an inverted microscope at ×400 magnification.

### 2.7. Cellular Cholesterol Contents

The collected cells were washed with cold PBS, and the free cholesterol (FC) and total cholesterol (TC) contents of the cells were quantified using a modified enzymatic fluorometric method. The lipid extracts were dissolved in isopropanol and then incubated with an enzyme mixture at 37°C for 1 h (for FC) or 2 h (for TC), followed by the addition of 0.1 M NaOH for 30 min to terminate the reaction. Fluorescence intensity was measured at excitation and emission wavelengths of 320 nm and 407 nm, respectively. Values were obtained from the calibration curves of standard FC and cholesterol ester (CE) for FC and TC, respectively. The concentration of CE was calculated by subtracting FC from TC. Standard curves were constructed in each set of experiments.

### 2.8. Western Blot Analysis

The cells were lysed in lysis buffer (Cell Signaling Technology, Danvers, MA) containing protease and phosphatase inhibitors. The proteins were measured using a DC protein assay kit (Bio-Rad Laboratories), resolved by NuPAGE Bis-Tris electrophoresis, and transferred onto nitrocellulose membranes (Amersham Biosciences). The cell membranes were blocked for 2 h in TBS containing 0.05% Tween 20 (TBST) and 5% nonfat milk powder. The blots were probed with rabbit antibodies against LOX-1 (1 : 1000), CD36 (1 : 1000), SR-B1 (1 : 1000), RAGE (1 : 1000), Gal-3 (1 : 1000), and *β*-actin (1 : 2000) and then were incubated with horseradish peroxidase-conjugated goat anti-rabbit secondary antibody (Sigma). The proteins were visualized using ECL western blotting detection reagents (GE Healthcare) and quantified by densitometry using ImageJ software. Density measurements were then normalised to *β*-actin readings.

### 2.9. Reverse Transcription-Quantitative Polymerase Chain Reaction (RT-qPCR) Analysis

Total RNA was extracted using TRIzol® reagent according to the manufacturer's instructions. Total RNA was reverse-transcribed into cDNA using Thermo Fisher RT reagents, and RT-qPCR was performed using SYBR Premix Ex Taq II, with gene-specific primers (synthesised by Sangon), on a Roche LightCycler® 96 System. The primers used for different target mRNAs are listed in Supplemental Table 1.

### 2.10. Statistical Analysis

The data were presented as the means ± S.D., and SPSS 17.0 software was used to analyse the data. Two-variable comparisons were analysed using unpaired Student's *t*-test. Multiple treatment-group comparisons were assessed by one-way ANOVA followed by a post hoc LSD test. *p* < 0.05 was considered statistically significant.

## 3. Results

### 3.1. Diabetic Amputation Patients Have Noticeable Plaques in Blood Vessels, and VSMCs in the Plaques Show a Macrophage-Like Phenotype

**Figure 1:**
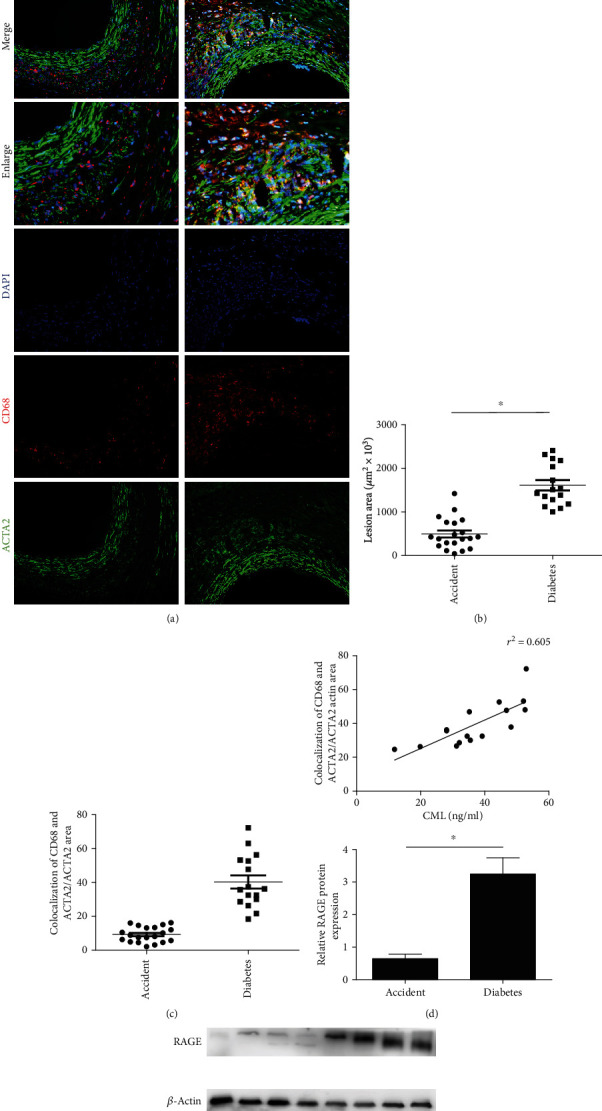
Compared with control patients, there is significant plaque enlargement and macrophage-like smooth muscle cells in the blood vessels of diabetic-amputated patients. (a) HE station and fluorescence statin show CD68 staining (red), ACTA2 (green), and DAPI (blue) from diabetic foot amputation or accident foot amputation. (b) Analysis of colocalization area of CD68 and ACTA2 shows orange in the pictures. (c) Comparison of plaque area in diabetic patients and control patients. (d) Comparison of colocalization of CD68 and ACTA2/ACTA2 area. (e) The expression of RAGE in the plaque. The averages of three serial cross-sections were used as single data points. ^∗^*p* < 0.05 compared with the control group.

Tissue specimens and serological samples from 16 patients with diabetic foot amputation were collected at the Affiliated Hospital of Jiangsu University. Compared with healthy control subjects, patients with diabetic foot amputation showed increased FPG, 2-HPG, and HbA1c (BMI) (Table 1). HE staining showed that diabetes patients have larger atherosclerosis plaques than healthy control subjects, and the staining of SMC-associated genes (ACTA2) and macrophage phenotype-related genes (CD68) in tissue sections from patients with diabetic foot amputation showed that the staining was colocalized in approximately 54% of the VSMCs in the plaques (Figures 1(a)–1(c)). Pearson correlation analysis indicated that the serum CML levels were correlated with the area of colocalization of CD68 and ACTA2 (Figure 1(d)), and western blotting showed that the expression of RAGE in vascular samples from patients with diabetic foot amputation was higher than that in the control group (Figure 1(e)). These results indicate that the advanced glycation end products can transfer from a smooth muscle cell phenotype to a macrophage-like state.

### 3.2. CML Induces the Formation of Vascular Smooth Muscle Cell-Derived Foam Cells

**Figure 2:**
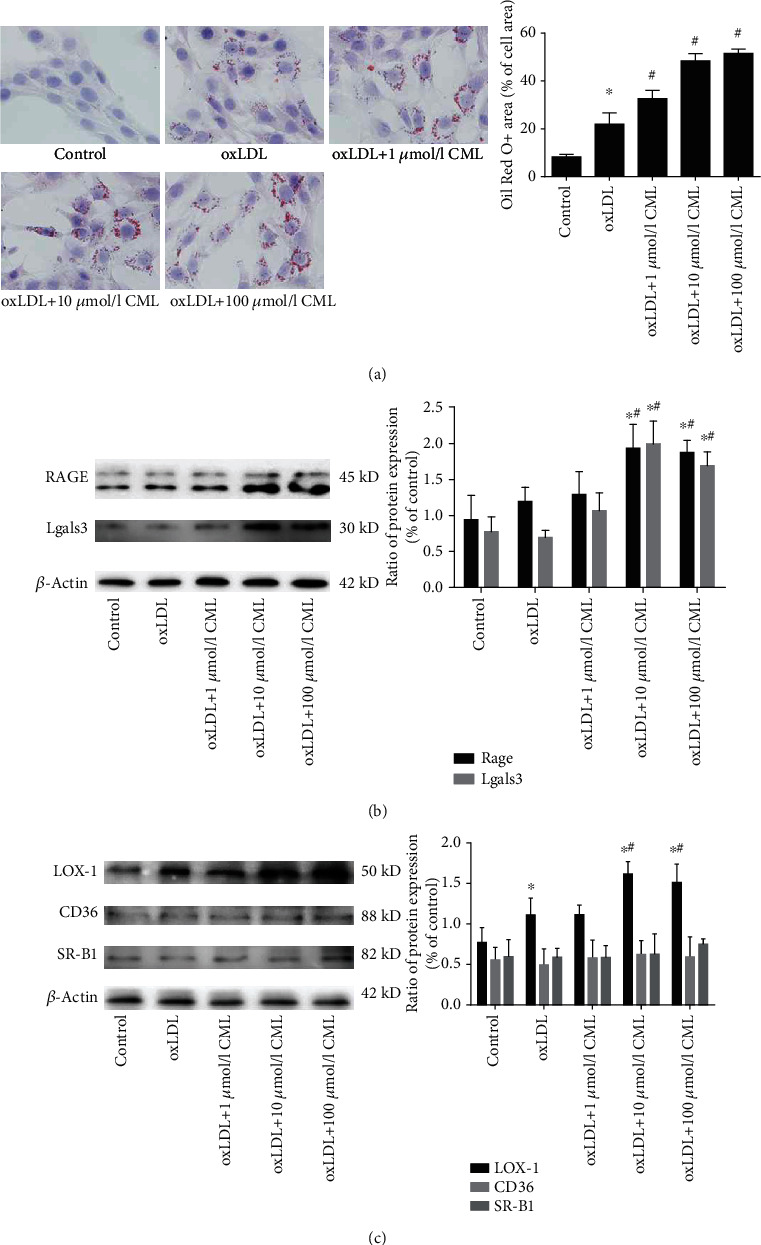
CML promotes lipid accumulation in VSMCs and increases RAGE and LOX-1 expression. (a) Primary VSMCs were incubated for 72 hours with indicated concentrations of oxLDL or prestimulated by CML. Cells were then fixed with 4% paraformaldehyde and stained with Mayer hematoxylin and oil red O. Magnification ×200. ^∗^*p* < 0.05 vs. control group, ^#^*p* < 0.05 vs. oxLDL group. (b) Proteins were extracted from primary VSMCs stimulated as above. Representative protein expression of receptors of CML RAGE and Gal-3, ^∗^*p* < 0.05 vs. control group, ^#^*p* < 0.05 vs. oxLDL group. (c) Representative protein expression of scavenger receptors CD36, SR-B1, and LOX-1, ^∗^*p* < 0.05 vs. control group, ^#^*p* < 0.05 vs. oxLDL group. Results are representative of 3 experiments with similar results.

To investigate the effects of AGEs on lipid accumulation in VSMCs, we pretreated VSMCs with a concentration gradient (0, 1, 10, and 100 *μ*mol/l) of CML for 6 h and then incubated the cells with 50 *μ*g/ml of oxLDL for 72 h. Oil red O staining showed that CML accelerated lipid accumulation in VSMCs in a dose-dependent manner (Figure 2(a)). The total cholesterol, cholesterol ester, and free cholesterol levels in VSMCs were increased significantly in the CML-pretreatment group than those in the oxLDL group. Notably, 100 *μ*mol/l of CML promoted an increase in the free cholesterol content, and the proportion was greater than 50%, suggesting that high concentrations of CML may have toxic effects on VSMCs (Table 2).

Next, we detected the expression of the main receptors of AGEs and scavenger receptors by western blotting and found that RAGE and Gal-3 expression levels were increased in a CML concentration gradient (Figure 2(b)). However, compared with the increased expression of CD36 and SRA1 in macrophage-lipid loading, we found no change in CD36 expression in SMC and the expression of LOX-1 increased with higher concentrations of CML, while the SR-B1 level was significantly increased only at 100 *μ*mol/l CML (Figure 2(c)).

### 3.3. CML Promotes Smooth Muscle Cell Foam Cell Formation by Upregulating RAGE Expression

**Figure 3:**
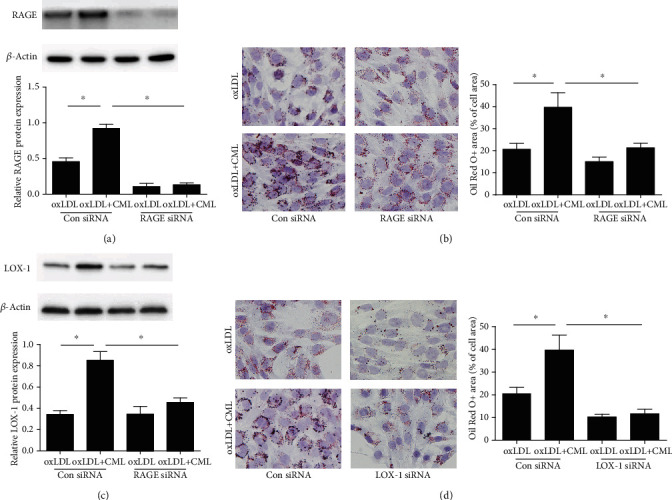
RAGE is essential for CML-induced lipid accumulation in VSMCs. Predesigned siRNAs for LOX-1 or RAGE were transfected to primary VSMCs at 25 nmol/l. VSMCs were used for experiments 72 h posttransfection and then treated by oxLDL or oxLDL and CML. (a) Representative western blot images of LOX-1. Results are representative of 3 experiments with similar results. ^∗^*p* < 0.05. (b) Oil red O staining of primary VSMCs transfected RAGE siRNA or negative control. Magnification ×200. ^∗^*p* < 0.05. (c) Representative western blot images of LOX-1. Results are representative of 3 experiments with similar results. ^∗^*p* < 0.05. (d) Oil red O staining of primary VSMCs transfected LOX-1 siRNA or negative control. Magnification ×200. ^∗^*p* < 0.05.

RAGE and Gal-3 are the main receptors of advanced glycation end products, and existing studies have shown that RAGE mediates the uptake of modified cholesterol lipoprotein and that Gal-3 (Lgals3) is relatively involved with VSMC phenotypic transformation to the macrophage-like state. In the present study, we found that siRNA interference of RAGE expression diminished lipid accumulation in CML-induced VSMC foam cell formation (Figures 3(a) and 3(b)). However, the degree of foam cell formation did not change after treatment with Gal-3 siRNA (Supplement Figure 1), suggesting that CML promoted foam cell formation via RAGE. Consistent with the cholesterol loading level, RAGE siRNA significantly decreased the LOX-1 level (Figure 3(c)). However, LOX-1 siRNA treatment significantly decreased the lipid accumulation capacity of VSMC, suggesting LOX-1 is an important receptor in the formation of VSMC-derived foam cells.

### 3.4. CML Enhances the Effect of oxLDL on the Phenotypic Transformation of Vascular Smooth Muscle Cells to a Macrophage-Like State

**Figure 4:**
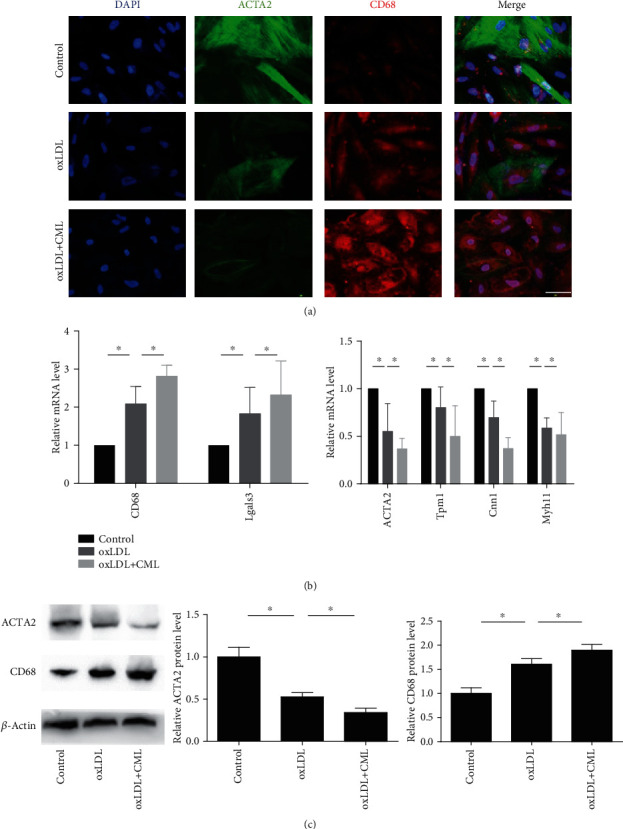
CML promotes phenotypic transformation in VSMC to a macrophage-like state. Primary VSMCs were incubated for 72 hours with indicated concentrations of oxLDL or prestimulated by CML. (a) Fluorescence photomicrographs show CD68 staining (red), ACTA2 (green), and DAPI (blue) in different groups. Magnification ×400. (b) Representative mRNA levels of macrophage phenotype and VSMC phenotype. Results are representative of 3 experiments with similar results. ^∗^*p* < 0.05. (c) Representative western blot images of ACTA2 and CD68. Results are representative of 3 experiments with similar results. ^∗^*p* < 0.05.

Existing studies have shown that cholesterol-loaded VSMCs lose the contractile phenotype and express a macrophage-like phenotype. We performed immunofluorescence staining, RT-PCR, and western blotting to investigate the effects of CML on VSMC phenotype transformation. The expression of the VSMC marker ACTA2 and increased expression of the macrophage-associated marker CD68 were decreased in the oxLDL group. Compared with oxLDL stimulation alone, CML prestimulation further reduced ACTA2 expression and increased the expression of CD68 (Figures 4(a) and 4(b)). Real-time qPCR was used to examine the expression of VSMC-related genes and macrophage-associated genes and showed that oxLDL decreased the mRNA levels of the VSMC-associated genes ACTA2, alpha-tropomyosin (Tpm1), smooth muscle myosin heavy chain (Myh11), and calmodulin (Cnn1), whereas the mRNA expression levels of the macrophage markers CD68 and Mac-2 (Lgals3) were significantly increased (Figure 4(c)). Pretreatment with CML enhanced the changed expression of phenotypic-related genes. These data indicate that CML enhances 1the effect of oxLDL on the phenotypic transformation of vascular smooth muscle cells to a macrophage-like state.

### 3.5. CML Converts VSMCs to a Macrophage-Like State by Upregulating RAGE Expression

**Figure 5:**
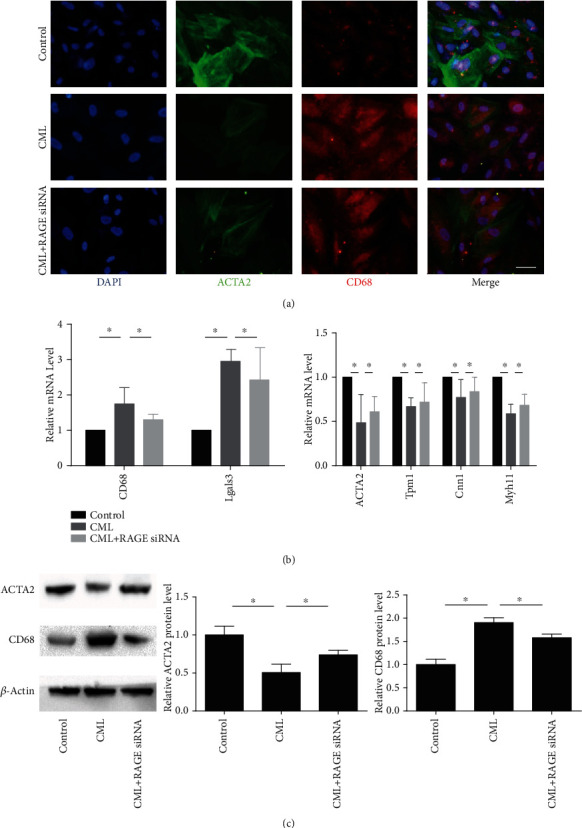
RAGE is essential for phenotypic transformation induced by CML in VSMC. Predesigned siRNAs for negative control or RAGE were transfected to primary VSMCs at 25 nmol/l. VSMCs were used for experiments 72 h posttransfection. (a) Fluorescence photomicrographs show CD68 staining (red), ACTA2 (green), and DAPI (blue) in different groups. Magnification ×400. (b) Representative mRNA levels of macrophage phenotype and VSMC phenotype. Results are representative of 3 experiments with similar results. ^∗^*p* < 0.05. (c) Representative western blot images of ACTA2 and CD68. Results are representative of 3 experiments with similar results. ^∗^*p* < 0.05.

In the above results, we demonstrated that CML promotes smooth muscle cell foam cell formation and enhances the effect of oxLDL on the phenotypic transformation of VSMCs. To further address the role of CML in VSMC phenotype transformation, VSMCs were stimulated by 10 *μ*mol/l of CML alone. CML treatment markedly increased the level of CD68 and decreased the expression of ACTA2. RAGE siRNA diminished CML-induced VSMC phenotype transformation (Figures 5(a) and 5(b)). The decreased expression of VSMC-related markers and increased expression of macrophage-related markers were reversed by RAGE siRNA (Figure 5(c)). These results show that CML converts VSMC to a macrophage-like phenotype, an effect mediated by RAGE.

### 3.6. CML Promotes the Functional Transformation of Smooth Muscle Cells into a Macrophage-Like State

**Figure 6:**
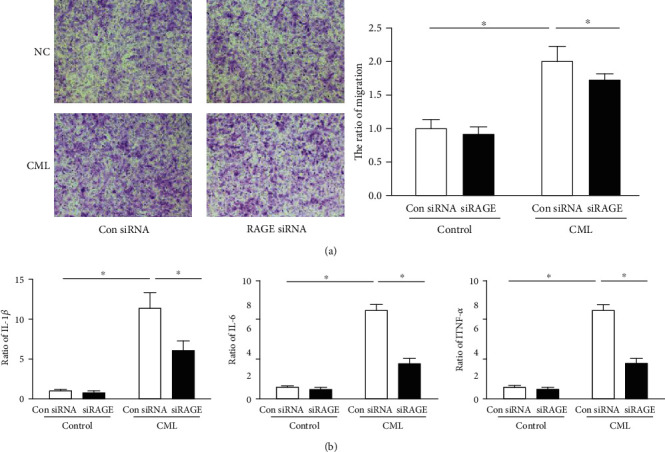
Effect of CML on VSMC secretion and migration. Predesigned siRNAs for negative control or RAGE were transfected to primary VSMCs at 25 nmol/l. VSMCs were used for experiments 72 h posttransfection. (a) Migration of VSMC induced by CML. Magnification ×200. (b) The level of IL-1*β*, IL-6, and TNF-*α* secreted from primary VSMCs. *n* = 4; ^∗^*p* < 0.05.

In previous experiments, we found that VSMCs that have undergone a phenotypic transformation to macrophages exhibit macrophage functions, but the functions were defective. To investigate whether CML can promote the functional transformation of VSMCs into macrophages, we examined the migration and inflammatory factor secretion abilities of VSMCs after CML stimulation. CML promoted the migration of SMCs (Figure 6(a)) and stimulated the secretion of IL-1*β*, IL-6, and TNF-*α* inflammatory factors (Figure 6(b)). We further treated the cells with RAGE siRNA and found that cell migration decreased and the secretion of inflammatory factors was also decreased. Therefore, we believe that CML can promote the functional transformation of SMCs into macrophages and that this process is mediated by RAGE.

## 4. Discussion

AGEs are heterogeneous groups of irreversible products resulting from the nonenzymatic glycation and oxidation of carbohydrates, proteins, nucleic acids, and lipids [17, 18]. AGEs are expressed in diabetes and induce cardiovascular disease complications [15]. In our study, we found noticeable plaques in the blood vessels of patients with diabetic amputation and observed a considerable amount of inflammatory cell infiltration by H&E staining. The inflammatory cells in plaques were detected by immunofluorescence staining of tissue sections from patients with diabetic amputation. Moreover, many VSMCs express macrophage markers in the infiltrating site, suggesting that macrophages in plaques are transformed from SMCs, a finding that is consistent with a previous study by Allahverdian et al. [7]. We detected the expression of CML and RAGE in serum and tissues and found that the levels of CML and RAGE in diabetic amputation patients were significantly increased. Pearson correlation analysis showed a significant correlation between serum CML and smooth muscle content in the vascular phenotype. However, CML promotes lipid accumulation in VSMCs cultured in vitro. Importantly, we report for the first time that CML can independently promote the transformation of VSMCs into macrophages, indicating the expression of SMCs in diabetic vascular injury. The disorder of intravascular metabolism plays a large role in this process.

The role of SMCs in atherosclerosis has been studied for decades, and recent studies have shown that VSMCs uptake lipid [19, 20] and undergo phenotypic transfer to a macrophage-like state in atherosclerotic plaques [7, 8, 21]. Existing studies have described that lipid-dependent macrophage uptake during atherosclerosis occurs via two pathways. In the first pathway, cells uptake different forms of modified lipoprotein, such as oxLDL, acetylated LDL, and ELDL via scavenger receptors [22–24]. The second pathway involves LDLr-mediated phagocytosis and receptor-independent pathways, such as macropinocytosis [25]. Chellan's team showed that ELDL promotes VSMC-derived foam cell formation via RAGE [26, 27]. In RAGE-/- VSMCs, ELDL stimulation of oxLDL uptake is significantly inhibited. Studies have shown that AGEs promote macrophage foam cell formation by inducing the uptake of oxLDL via two important oxLDL receptors, CD36 and SR-B1 [28]. Our experiments demonstrated that increased CML promotes foam cell formation, which is mediated by RAGE. Notably, the scavenger receptors CD36 and SR-B1 did not play a role in this process, while another scavenger, LOX-1, was significantly upregulated. After the treatment of VSMCs with RAGE siRNA and LOX-1 siRNA, the degree of lipid accumulation in VSMC significantly decreased, suggesting that CML-induced VSMC foam cell formation is mediated by LOX-1, but studies of the specific underlying mechanism are not included in this paper.

Our results have several clinically relevant implications. By counting the number of SMCs that transformed into a macrophage phenotype in vascular sections of diabetic-amputated patients and measuring the RAGE concentration in blood vessels and serum CML levels, we found that the CML level and the phenotype-switched vascular smooth muscle content are correlated, which is a highly significant finding. Because previous studies have reported that VSMCs convert to the macrophage phenotype after cholesterol loading [7, 19, 29, 30], our study revealed the possibility that the advanced glycation end products can also promote VSMCs. Notably, in the case of transformation into macrophages, the individual effects of CML and oxLDL were not increased by costimulation with CML and oxLDL, suggesting a possible competitive relationship between CML and oxLDL.

Unlike macrophages, VSMCs in the vessel wall usually do not have inflammatory properties, whereas inflammatory responses often occur in vascular smooth muscle and play an important role in many cases of atherosclerosis [31, 32]. CML promotes inflammatory responses and ROS production in SMCs, activities that are important in lipid metabolism. In this study, we found that CML promotes the secretion of IL-1*β*, IL-6, and TNF-*α* by SMCs. Consistent with previous studies, the secretion of inflammatory factors was significantly reduced after RAGE siRNA interference, and more data are needed to clarify the relationship between inflammation and the SMC phenotype.

To summarize, we explored another possible line of SMC phenotypic transformation in diabetic patients in this study. In our study, the glycation end product CML was found to promote lipid accumulation in SMCs. Furthermore, by inhibiting RAGE and Gal-3, we discovered the role of RAGE in this process. On the other hand, CML promotes the inflammatory response in SMCs and the phenotypic transformation to macrophages, providing a new perspective on a pathological phenomenon to diagnose diabetic vascular injury.

## Figures and Tables

**Table 1:** Baseline characteristics and laboratory data of the studied population.

	DM *n* = 16	Non-DM *n* = 20	*p*
	Mean ± SD	Mean ± SD	
Age (years)	70.13 ± 10.74	68.52 ± 8.36	0.61
Duration of diabetes (years)	14 ± 3.81	—	—
Male/female	9/7	11/9	—
FPG (mmol/l)	7.86 ± 2.13	5.05 ± 0.32	<0.001
2-HPG (mmol/l)	13.82 ± 4.06	5.52 ± 1.13	<0.001
HbA1c (%)	7.99 ± 1.84	5.74 ± 0.49	<0.001
Cholesterol (mmol/l)	3.95 ± 1.17	4.43 ± 0.67	0.13
LDL (mmol/l)	2.31 ± 1.05	2.61 ± 0.71	0.31
HDL (mmol/l)	0.91 ± 0.48	1.02 ± 0.33	0.499
Triglycerides (mmol/l)	1.43 ± 0.82	1.84 ± 1.03	0.203
Creatinine (*μ*mol/l)	82.02 ± 19.2	78.9 ± 15.2	0.589
BMI (kg/m^2^)	28.92 ± 3.16	27.84 ± 4.01	0.3850
Smoking (*n*)	7	9	—
Oral antidiabetic agents (*n*)	15	0	—
Insulins (*n*)	10	0	—
Lipid-lowering therapy (*n*)	6	3	—
Antihypertensive therapy	4	5	—
CML (ng/ml)	48.80 ± 7.31	13.12 ± 3.20	<0.001

**Table 2:** Lipid levels in VSMC stimulated by different concentrations of CML.

Treatment	Mouse VSMC cholesterol mass (*μ*g/mg protein)			
		CE (% of TC)	FC	TC
No oxLDL		6.64 ± 1.14 (12.2%)	46.5 ± 4.85	52.97 ± 5.72
oxLDL-loaded				
CML	None	35.86 ± 6.48^a^ (36.6%)	62.1 ± 9.8^a^	97.97 ± 16.26^a^
	1 *μ*mol/l	42.27 ± 5.79 (41.5%)	67.37 ± 22.57	113.64 ± 28.11
	10 *μ*mol/l	101.93 ± 19.55^b^ (52.3%)	78.2 ± 6.2	180.13 ± 17.41^b^
	100 *μ*mol/l	139.3 ± 15.83^b^ (48.0%)	150.37 ± 14.59^b^	289.67 ± 24.13^b^

^a^ *p* < 0.05, compared with the no oxLDL group, ^b^*p* < 0.05, compared with the oxLDL-loaded group.

## Data Availability

The data used to support the findings of this study are included within the article.

## Supplementary Materials

Supplementary Materials Table S1: RT-PCR primers of VSMC-related gene and macrophage-related gene. Table S2: subject characteristics, primary diagnosis, and percent SMC foam cells. Supplemental Figure 1: Gal-3 siRNA did not change the degree of CML-induced lipid accumulation in VSMC.
