# Supplementary material for: Advanced Glycation End Products Induce Vascular Smooth Muscle Cell-Derived Foam Cell Formation and Transdifferentiate to a Macrophage-Like State
Source: Mediators Inflamm. 2020 Aug 7;2020:6850187. doi: 10.1155/2020/6850187 (PMC7428884; doi:10.1155/2020/6850187)

Supplemental Table 1. RT-PCR primers of VSMC related gene and Macrophage related gene

Gene	Directin	Primer Sequence
Cnn1	For	GGACCAGGCGACCATCAG
	Rev	TAGGCAGAGTTGTAGTAGTTGTG
Acta2	For	GCTTCGCTGGTGATGATGCTC
	Rev	AGTTGGTGATGATGCCGTGTTC
Myocd	For	AAGGTCCATTCCAACTGCTC
	Rev	CCATCTCTACTGCTGTCATCC
Gapdh	For	CGTGCCGCCTGGAGAAAC
	Rev	TGGGAGTTGCTGTTGAAGTCG
CD68	For	CTTCCCACAGGCAGCACAG
	Rev	ATGATGAGAGGCAGCAAGAGG
Lgals3	For	AGGAGAGGGAATGATGTTGCC
	Rev	GGTTTGCCACTCTCAAAGGG

Supplemental Table 2. Subject characteristics, primary diagnosis and percent SMC foam cells

Subject no.	Age(years)	Sex	Primary diagnosis	Percent of SMC foam cells
1	61	F	diabetic foot, type 2 diabetes	72.3
2	67	F	Right lower limb amputation wound ulcer, type 2 diabetes	37.4
3	75	M	Left lower limb erysipelas, diabetic foot, type 2 diabetes	26.3
4	81	M	diabetic foot, type 2 diabetic	21.6
5	67	F	Left diabetic foot, type 2 diabetes	52.7
6	62	M	Left diabetic foot, type 2 diabetes	18.4
7	70	F	Right diabetic foot, type 2 diabetes	53.2
8	79	M	Right diabetic foot, type 2 diabetes	35.7
9	74	M	Left foot ulcer, diabetic foot disease, radioactive dermatitis type 2 diabetes	28.6
10	62	F	Right diabetic foot, type 2 diabetes	30.1
11	71	M	Right diabetic foot, type 2 diabetes	63.1
12	69	F	Left diabetic foot, type 2 diabetes	32.5
13	74	M	Right diabetic foot, type 2 diabetes	47.8
14	68	M	Left diabetic foot, type 2 diabetes	56.3
15	72	F	Right diabetic foot, type 2 diabetes	36.2
16	58	M	Right diabetic foot, type 2 diabetes	32.5

Supplemental Figure 1. Gal-3 siRNA did not change the degree of CML induced lipid accumulation in VSMC

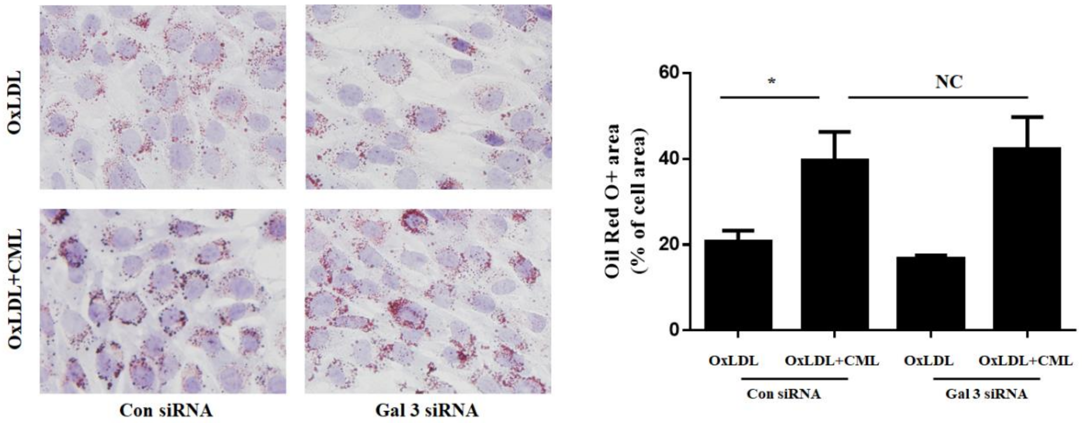

Supplement: Supplementary Materials — Table S1: RT-PCR primers of VSMC-related gene and macrophage-related gene. Table S2: subject characteristics, primary diagnosis, and percent SMC foam cells. Supplemental Figure 1: Gal-3 siRNA did not change the degree of CML-induced lipid accumulation in VSMC. [file 6850187.f1.pdf]
